# Spatiotemporal Dynamics of Virus Infection Spreading in Tissues

**DOI:** 10.1371/journal.pone.0168576

**Published:** 2016-12-20

**Authors:** Gennady Bocharov, Andreas Meyerhans, Nickolai Bessonov, Sergei Trofimchuk, Vitaly Volpert

**Affiliations:** 1 Institute of Numerical Mathematics, Russian Academy of Sciences, Moscow, Russian Federation; 2 Infection Biology Laboratory, Department of Experimental and Health Sciences, Universitat Pompeu Fabra, Barcelona, Spain; 3 ICREA, Pg. Lluís Companys 23, Barcelona, Spain; 4 Institute of Problems of Mechanical Engineering, Russian Academy of Sciences, Saint Petersburg, Russian Federation; 5 Instituto de Matemática y Fisica, Universidad de Talca, Talca, Chile; 6 Institut Camille Jordan, UMR 5208 CNRS, University Lyon 1, Villeurbanne, France; 7 INRIA Team Dracula, INRIA Lyon La Doua, Villeurbanne, France; 8 Laboratoire Poncelet, UMI 2615 CNRS, Moscow, Russian Federation; 9 Gamaleya Center of Epidemiology and Microbiology, Moscow, Russian Federation; 10 RUDN University, Moscow, Russian Federation; Shanxi University, CHINA

## Abstract

Virus spreading in tissues is determined by virus transport, virus multiplication in host cells and the virus-induced immune response. Cytotoxic T cells remove infected cells with a rate determined by the infection level. The intensity of the immune response has a bell-shaped dependence on the concentration of virus, i.e., it increases at low and decays at high infection levels. A combination of these effects and a time delay in the immune response determine the development of virus infection in tissues like spleen or lymph nodes. The mathematical model described in this work consists of reaction-diffusion equations with a delay. It shows that the different regimes of infection spreading like the establishment of a low level infection, a high level infection or a transition between both are determined by the initial virus load and by the intensity of the immune response. The dynamics of the model solutions include simple and composed waves, and periodic and aperiodic oscillations. The results of analytical and numerical studies of the model provide a systematic basis for a quantitative understanding and interpretation of the determinants of the infection process in target organs and tissues from the image-derived data as well as of the spatiotemporal mechanisms of viral disease pathogenesis, and have direct implications for a biopsy-based medical testing of the chronic infection processes caused by viruses, e.g. HIV, HCV and HBV.

## Introduction

### Biological background

Human infections with viruses such as HIV, hepatitis B and C viruses, influenza A virus, present enormous burden to public health worldwide. The defence against various pathogens including viruses is a major function of the immune system [1–3]. It is generally accepted that the outcome of a virus infection results from the “numbers game” characterized by the kinetics of virus growth in target cells, its spread across sensitive tissue and the strength of the antiviral immune responses [4, 5]. Recent advances in imaging and visualizing virus-specific T cells, cytokines and infected cells in living hosts [5–7] open new opportunities for developing a mechanistic quantitative understanding of the general regularities of the spatiotemporal dynamics of virus infections [8].

Viruses are obligatory parasites that need cells to replicate their genomes and produce progeny. Depending on the mode of transmission, a virus will expand locally around its entry site of a newly infected organism. From there it might subsequently spread to other tissues and organs, and a new transmission event to a new host can be initiated.

Amongst the first quantitative descriptions of virus spread within an organism is the work of Frank Fenner in the 1940ies who studied ectromelia virus infection of mice causing mousepox [9]. The virus titres over time were bell-shaped curves with varying maximum titres and widths within spleen, skin, and peripheral blood. This bell-shaped behavior is a reflection of virus expansion in available target cells and virus restriction from concomitantly induced immune responses. Elegant more recent work on Simian Immunodeficiency virus (SIV) in monkeys and Lymphocytic Choriomeningitis virus (LCMV) in mice provide details on this spreading process for these prototypes of non-cytopathic viruses [10, 11]. Virus infection and expansion activates the proliferation of virus-specific cytotoxic T lymphocytes (CTL). These cells recognize infected cells that present peptides from viral proteins in context with so-called major histocompatibility complex proteins on their surface. Target cell recognition then triggers the CTL to release lytic enzymes from intracellular granules. When infected cells are exposed to such enzymes, they are induced to die and thus, the centers of virus production are eliminated. Given this mechanistic scheme, it is obvious that the final outcome of an infection will be determined by the dynamic properties of (i) virus replication and spread to new target cells, and (ii) CTL-mediated target cell recognition and killing. In this context, Blancou et al [10] analyzed the time frames of localized antigen-induced SIV production and SIV-specific CTL infiltration demonstrating that the physiologically relevant window of virus spread to new target cells *in vivo* may only be few hours until CTL may clear an infectious center. Li et al visualized simultaneously virus-producing cells and CTL in tissue of SIV-infected Macaques and LCMV-infected mice [11]. They observed a direct correlation of virus reduction with increasing CTL effector to infected target cell ratios. Thus the extent of virus control seems directly related to the timing and magnitude of the virus-specific CTL response [3, 11].

### Spatiotemporal models of viral infections

Mathematical models have been extensively used to study the dynamics of viral infections and antiviral responses mostly under the simplifying assumption of spatial homogeneity, i.e. the host macro-organism is a well-mixed compartment or a small set of such compartments [12–14], with a few models considering the spatial spread of the viruses in infected hosts. The available spatially extended models of viral infection dynamics are briefly summarized in (S1 Table) and described below.

It was stated in [8] that viral propagation in HIV infection is a fundamentally local process because the virus is inherently unstable and the infection occurs mainly in lymphoid tissues. To study the spatiotemporal dynamics of HIV propagation, a three-dimensional stochastic cellular automata model was formulated. It describes the viral spread among a cluster of CD4+ T cells. The impact of biophysical parameters of the virus diffusion and inactivation on the basic reproductive ratio are discussed in detail for a low-density and high density of target cells. The model predicted the existence of distinct viral propagation regimes: a stable propagation as a traveling wave, a chaotic steady state in which infected, target and empty sites coexist, and extinction of infection due to insufficient target cell replenishment. The viral spread at low cell densities is limited by the stability of the free virus whereas at high densities it depends mostly on the geometry of the lattice, i.e. on the cell radius. The model is considered to be too crude to make detailed quantitative predictions for *in vivo* lymphoid tissues.

Data driven modelling of virus growth and spread is presented in [15]. The authors build a reaction-diffusion model to analyze the spatiotemporal patterns of virus infection spreading. The data characterize the vitro growth of vesicular stomatitis virus (VSV) in baby hamster kidney cells (BHK) quantified via immunohistochemical labeling and digital imaging. The model assumes a radial symmetry of the propagation and compares the description of virus production in infected cells with- and without accounting for the infection age. The impact of diffusible interferon (IFN) on the virus spread has also been examined. Data fitting was used to estimate a number of virus-target cell interaction parameters. The model reproduces the experimental data on the initial outward amplified propagation of the infection front followed by stagnation due to antiviral IFN responses.

The effects of spatial heterogeneity of infected cells and immune cells on the viral infection dynamics was examined in [16]. The development and outcome of influenza A virus infection (IAV) was modelled via a two-dimensional cellular automation model. Numerical simulations with the model were used to study the effect of the grouping of the infected cells as patches of various sizes and the longevity of the immune responses on the dynamics of infection. Depending on the rules of local vs global regeneration of epithelial cells, and the scenario for immune cell addition at random sites or at the infection site (recruitment), different regimes of the infection spread were predicted including the existence of circular waves of the infection spread in the upper respiratory tract tissue and the possibility of a low level persistence of the infection. A similar approach has been recently developed with a spatial consideration of the virus growth in the lung modelled as a two-dimensional sheet of hexagonally-tilled epithelial cells in [17].

The impact of spatial structure on the dynamics of virus infections has been studied in [18] using a multi-compartment description of the organ tissue in which the infection takes place. In fact the spatial organization of the organ was represented by two-dimensional square grid with 21x21 sites in which target and infected cells were sessile. At each site the population dynamics of the virus infection and the T cell response was described by a system of ODEs. The tissue environment was called a homogeneous one when the parameter values were identical for each site. Otherwise, it was called heterogeneous. The local diffusion was modelled by the possibility of the virus to randomly spread from site to site of the grid. The results of the modelling suggest a number of important effects of the spatial extension of the model. For example, the spatial coupling of the sites by local dispersal of the virus reduced the amplitude of the oscillation in the viral load dynamics. This implied a more stable persistence of the infection *in vivo* and questioned the validity of the concept of the “dynamics elimination of pathogen”. Recently, a similar approach was used to study the dynamics of HIV infection in the network of lymphoid tissues [19].

In the case of HIV infection two modes of virus propagation take place: the local spread of HIV within lymphoid tissues, where target cells (CD4+ T lymphocytes) are densely packed and the hematogeneous spread of virus to distant lymphoid tissues and organs. The implications of the two propagation processes for the dynamics of HIV-1 infection were studied using mathematical models of cell-to-cell and cell-free viral spread [20]. They formulated a two-dimensional model of cell-to-cell spread using a system of distributed delay differential equations (DDE) with the eclipse phase described by a gamma distribution. The analysis of the model suggested that the delayed models of cell-to-cell spread produce sustained oscillations of infection for typical tissue culture parameters. This was in contrast to a typical ODE model of HIV infection spread via cell-free virus which predicted a globally asymptotically stable infection state. A positive link between the existence of latently infected cells and the persistence of HIV infection was proposed. A recent study of the relative role of the two spreading mechanisms of HIV-1 infection with a system of ODEs [21] suggests that an essential feature of HIV infection progression is a combination of cell-free infection following fluid-phase diffusion of viruses and direct cell-to cell-transmission at immune cell contacts (so called ‘hybrid spreading mechanism’). The model based analysis of real patients data revealed that the hybrid spreading of the virus is critical for the initial establishment of the infection whereas the cell-to-cell dissemination is important for disease progression.

The early stage of sexual transmission of HIV through the epithelium is characterized by a sporadic and irregular establishment of single foci of infection. To understand the possibility of emergence of spatial heterogeneity in the spread of HIV infection due to diffusion and chemotaxis of target cell rather than due to underlying spatial heterogeneity of the tissue, a reaction-chemotaxis -diffusion model was proposed in [22]. This model was formulated for a two-dimensional representation of the epithelial surface. Virus, target cell and infected cells were assumed to follow a purely Brownian motion. The emergence of the hot spots of infection, their spatial frequency and the speed of propagation were examined in relation to various parameters of the model as well as the initial distribution of the target cells. The necessary conditions for Turing instability were obtained analytically and further explored in numerical simulations. It was shown that HIV infection does not need a spatially heterogeneous tissue structure for the formation of spatial patterns in the form of hot spots. Importantly, it was proposed that the immune response to HIV may give rise to pattern formation. The modelling results have implication for the application of microbicides against HIV.

Some infections are characterized by the transport of virus and immune cells to the inner parts of susceptible solid organs through their surfaces. A mathematical model of viral infection inside a spherical organ was formulated in [23]. The model variables represent stationary cell densities and transported quantities: viruses, humoral factors and immune cells. The model assumes that the mobile quantities penetrate from the organ surface from an external source via diffusion. The population dynamics of the viral infection is formulated by a system of reaction-diffusion equations with boundary conditions specified at the surface of the sphere and its center (radial symmetry). Numerical simulations were used to explore the effect of biophysical parameters on the distribution of viruses, immune cells and antiviral drugs inside the organ. It was shown that spatial heterogeneity of the organ leads to a gradient in the steady state distribution of the virus inside the organ and to a damped oscillations in the viral load assuming that immune cells proliferate outside the organs. The model extensions were formulated to include latently infected cells and diffusion of antiviral drugs inside the organ. These were used to examine the dependence of the viral distribution on the diffusivity parameters of the mobile components and compared to some data from HIV infections.

To identify the mechanism and parameters underlying the local growth of hepatitis C virus (HCV) *in vitro* a data-driven approach was developed in [24]. The kinetics of the infected cells in foci was quantified by immunohistochemical staining over a period of 72 hours. The model for the cell-to-cell transmission of the virus was formulated as a master equations system. The data on HCV focus expansion were best described by a model assuming a focus size-dependent growth rate. Finally, there exist few hybrid approaches that model the immune response in lymph nodes using an agent-based framework for cell dynamics and the spatiotemporal description of cytokines and antigens via reaction-diffusion equations [25–28]. A two-dimensional lattice approximation of the permissive organs is used. Although the descriptive potential of hybrid models is high, their practical applications are still to come due the enormous computational complexity and uncertainty in the parameter values. The later aspect creates a danger of generating modelling artefacts whereas the complexity of the models limits their applicability in providing a global view of the spatiotemporal dynamics of viral infections in the face of antiviral immune responses.

The models reviewed above have one feature in common: the antiviral immune reaction is considered (if any) following the predator-prey framework, in the sense that immune cells multiply due to the presence of infection and eliminate it. However, it is broadly accepted now that viruses can downregulate the specific immune cells via their physical or functional exhaustion mechanisms thus leading to the establishment of chronic infectious diseases. Hence prey is not only consumed by predators but it can also hunt predators [2, 29, 30]. Straightforward examples are provided by HIV, HBV and HCV infections in humans [31–33] and experimental LCMV infection in mice [34–37]. In this paper we propose a model of spatiotemporal virus infection dynamics which considers a non-linear bell-shaped regulation of the cytotoxic T cells response with a time lag needed for their clonal expansion. The model is used to provide an analytical insight into the regulation of various patterns of viral infection dynamics. Numerical experiments are conducted to link the predicted spatiotemporal regimes with empirical observations. Finally, we discuss the modelling results and make conclusions.

## Mathematical model

**Fig 1:**
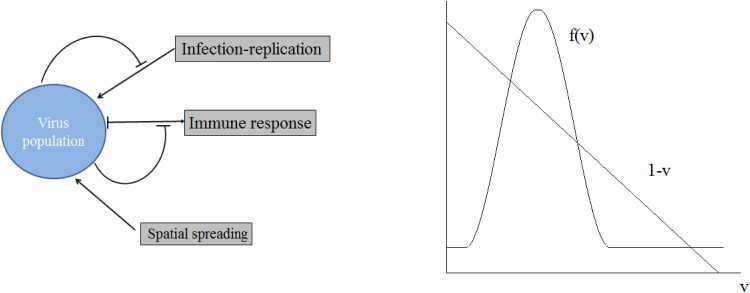
Schematic representation of virus infection dynamics regulation (left) and qualitative forms of the function *f*(*v*) (right). Low level infection stimulates immune response while high level infection down-regulates it. The former corresponds to the growing branch of the function *f*(*v*) while the latter to its decreasing branch.

To formulate the equation of the virus dynamics, we follow the view that antigen dose, time period during which it is available and its “geographical” distribution within this host influence the duration and extent of the immune responses [38]. The underlying regulation implies a bell-shaped relationship between viral load and the magnitude of the antiviral T cell response so that high antigen amount leads to exhaustion of T cells [39, 40]. The biological scheme of the model and the relevant processes are shown in Fig 1(a). To make the model analytically tractable, we do not consider explicitly the population dynamics of the antiviral T cell response. The magnitude of the clonal expansion is assumed to be a non-linear function of the viral load with a time lag. The common practice of using time delay in the life sciences is when there are some hidden variables and processes which are not well understood but are known to take some time to react. Although the model is general enough, we develop it with a particular interest to study non-, or weakly cytopathic infections such as LCMV, SIV and HIV-1.

We consider the following equation for the concentration of virus *v* in lymphoid organs such as the spleen or lymph nodes or in any other permissive tissues:

$$\frac{\partial v}{\partial t}=D\frac{\partial^{2}v}{\partial x^{2}}+kv\left(1-v\right)-f\left(v_{\tau }\right)v.$$ (1)

Here *v* = *v*(*x*, *t*), *v*_*τ*_ = *v*(*x*, *t* − *τ*), the function *f*(*v*_*τ*_) will be specified below. The first term in the right-hand side of this equation describes virus diffusion, the second term its production and the last term its elimination by CTL cells. The parameter *D* is the diffusion coefficient (or diffusivity) and *k* stands for the replication rate constant. The parameterized function *f*(*v*_*τ*_) characterizes the virus induced clonal expansion of T cells, i.e. the number and function of these cells generated after some time-delay *τ*, i.e. it depends on the concentration of virus some time before. The qualitative form of this function is shown in Fig 1(b) [41].

Eq (1) is a quasi-linear functional reaction-diffusion equation. The fundamentals of the general theory of these equations can be found in [42, 43]. It is worthwhile to note that for *t* ∈ [*t*_0_, *t*_0_ + *τ*] each solution of Eq (1) satisfies the usual non-autonomous reaction-diffusion equation

$$v_{t}=v_{xx}-a\left(t,x\right)v+kv\left(1-v\right),\;t\geq t_{0}$$ (2)

with some boundary conditions and with the initial condition *v*(*t*_0_, *x*) = *v*_0_(*t*_0_, *x*), where *v*_0_(*s*, *x*), *s* ∈ [*t*_0_ − *τ*, *t*_0_] is the prescribed initial function for Eq (1) and *a*(*t*, *x*) = *f*(*v*_0_(*t* − *τ*, *x*)). After solving Eq (2) on [*t*_0_, *t*_0_ + *τ*] we can again apply the same argument to (1.1) for *t* ∈ [*t*_0_ + *τ*, *t*_0_ + 2*τ*] and so on. This procedure is known as the method of steps [44], it is simple and efficient but it works only on finite time intervals. Consequently, other approaches should be applied in order to study global in time behaviour of solutions to Eq (1). One can expect (and this is confirmed analytically, cf. [44]) that the small delays are “harmless” in the sense that the asymptotic properties of solutions of delay differential equations varies continuously as *τ* → 0+. However, if *τ* becomes large, the delayed nonlinearity might trigger essential qualitative changes in the dynamics of the system. It also presents a considerable technical complication from the mathematical point of view.

In this respect, the delayed reaction-diffusion equations whose reaction term *g* is either of logistic type (i.e. *g* is as in (1.1), when *v* is not separated multiplicatively from *v*_*τ*_) or of the Mackey-Glass type (when *v* is separated multiplicatively from *v*_*τ*_, i.e. *g* = −*kv* + *b*(*v*_*τ*_)) are between the most studied ones. It is an interesting point of discussion whether the Mackey-Glass type models reflect more adequately the biological reality than the logistic models, see e.g. [45] and especially [44] for further details. In any case, in many regards both models exhibit similar types of qualitative behaviour of solutions. In particular, this can be seen from the existence, uniqueness and stability results describing travelling waves (both monostable and bistable) for various subclasses of the logistic type and the Mackey-Glass type equations, see [1, 46–52] among many other references. We note that the investigation of delayed logistic models is more difficult and technically involved than the studies of the Mackey-Glass type systems, precisely because of the multiplicative non-separateness of *v* and *v*_*τ*_. For example, so far no analytical results on the existence and uniqueness of monostable (or even bistable, cf. [53–58]) waves in the delayed Eq (1) with *non-monotone nonlinear* response *f* were available in the literature. This work presents the first contribution in this respect: below, we propose a novel approach to the both aforementioned problems. This approach combines the phase plane method with simple fixed points arguments.

We will also study nonlinear dynamics of reaction-diffusion waves described by Eq (1). Travelling waves in populations dynamics are extensively studied in ecology and epidemiology (see [59–61] and the references therein). In the case of reaction-diffusion systems they can be accompanied by pattern formation and spreading [59, 62–65]. Pattern formation and wave propagation in the case of delay reaction-diffusion equations are discussed in [66–68].

## Virus spread without time delay

The above model is used to identify and characterize some fundamental types and patterns of the spatiotemporal dynamics of virus infections in tissues. We start the analysis of the model with a simplifying assumption of *τ* = 0. In this case, virus distribution in the tissues such as spleen or lymph node can be described by the reaction-diffusion equation

$$\frac{\partial v}{\partial t}=D\;\frac{\partial^{2}v}{\partial x^{2}}+kv\left(1-v\right)-f\left(v\right)v.$$ (3)

The above model governs a spatio-temporal dynamics of the virus infection resulting from a balance of the diffusion, replication, and elimination by the cytotoxic T cell response. The T cell response depends in a bell-shaped way on the viral load. In contrast to Eq 1, the time needed for the cells to expand and migrate to the site of infection is assumed to be taken into account indirectly via the characteristics of the function *f*(⋅) rather that explicit time delay *τ*. Here *v* = *v*(*x*, *t*) is the dimensionless normalized virus concentration, *x* ∈ *R*_+_, *t* ∈ [0, +∞). By a re-scaling of variables this equation can be reduced to the same equation with *D* = 1, *k* = 1. We will consider this equation on the whole axis, i.e., in Ω = ((*x*, *t*): −∞ < *x* < +∞, 0 ≤ *t* < +∞) with a non-negative initial condition *u*(*x*, 0) = *u*_0_(*x*).

For what follows it is convenient to introduce the function

$$F\left(v\right)=v\left(1-v-f(v)\right).$$ (4)

Depending on the form of the function *f*(*v*) we will get different behavior of solutions. Let us note that *F*(0) = 0. This function can have other zeros for *v* > 0. We will also suppose that the function *f*(*v*) is non-negative, it is continuous together with its second derivatives.

Under the above assumptions, this model of virus infection implies a natural description of the spatiotemporal dynamics of the virus in terms of travelling- or standing waves. The travelling wave represents a pattern in the viral density that travels across the tissue in an uninterrupted fashion. Another possibility represented by a standing wave would be a viral density pattern that appear to be confined to some region and standing still. Note that standing waves are the partial case of traveling waves correspond to a zero velocity of the wave front. Hence, we do not consider them as a separate case.

We will study the conditions for the existence and properties of travelling wave solution of Eq (3), that is solutions of the form *v*(*x*, *t*) = *w*(*x* − *ct*) that moves at a constant speed *c* in the positive *x*-direction. The function *w*(*x*) (we keep the same notation *x* for the argument of *w*(⋅)) satisfies the equation

$$w^{\prime \prime }+cw^{\prime }+F\left(w\right)=0$$ (5)

with appropriate conditions at infinity equal one of the steady state, i.e., lim_*x* → −∞_ *w*(*x*) = *v*_*i*_, lim_*x* → +∞_ *w*(*x*) = *v*_*j*_ *i*, *j* = 0, 1, 2, 3, *i* ≠ *j*. The constant *c* here is the wave speed and its relationship to original model parameters will be established by the analytical treatment. The value of *c* is a solution to the above eigenvalue problem.

**Fig 2:**
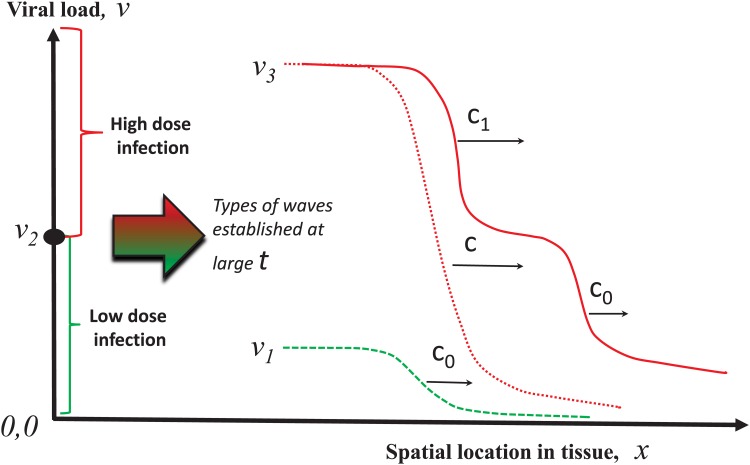
Schematic representation of the spatial patterns of virus infection dynamics as travelling waves. Typical wavefront solutions taking a steady state value *v*_*i*_ at the left end and another steady state *v*_*j*_, *i* ≠ *j* at the right end. The travelling waves evolve with the speed *c*. A qualitative relationship between the initial viral load and the emerging pattern on the spatiotemporal pattern of virus spread is sketched.

As shown in Fig 2, the model admits the existence of three types of regimes: (i) a small amplitude wave corresponding to a low-level infection spreading at constant *c*_0_ through the tissue, (ii) a large amplitude wave corresponding to a high-level infection spreading at constant *c*_1_ through the tissue, and (iii) a large amplitude two-wavefront solution corresponding to a low-level infection followed by a hight level one evolving at different wave speeds *c*_1_ and *c*_0_, respectively.

### Existence of waves

In this section we outline the conditions of the existence of three types of propagating solutions shown in Fig 2. Suppose that the equation

f(v)=1-v (6)

has three positive solutions *v*_*i*_, *i* = 1, 2, 3 as shown in Fig 1. Then the function *F*(*v*) has four zeros: *v*_0_ = 0, and *v*_1_, *v*_2_, *v*_3_ which are positive and ordered: *v*_1_ < *v*_2_ < *v*_3_.

1. The small amplitude monotonically decreasing solution of Eq (5) with the limits
  $$w\left(-\infty \right)=v_{1},\;\;\;w\left(\infty \right)=0$$ (7)
  is known to exist for all values of *c* ≥ *c*_0_, where *c*_0_ is a positive minimal wave speed [69, 70]. Non-monotone solutions can also exist for *c* < *c*_0_. However they are not positive and hence are not practically relevant.
2. The large amplitude solutions of Eq (5) with the limits
  $$w\left(-\infty \right)=v_{3},\;\;\;w\left(\infty \right)=v_{1}$$ (8)
  exist for a unique value *c* = *c*_1_.
3. The existence of two-wave solutions with the limits
  $$w\left(-\infty \right)=v_{3},\;\;\;w\left(\infty \right)=0$$ (9)
  depends on the values *c*_0_ and *c*_1_. If *c*_0_ ≥ *c*_1_, then such solutions do not exist, if *c*_0_ < *c*_1_, then they exist for all *c* such that c_*_ ≤ *c* < *c*_1_ with some c_*_ ≥ *c*_0_. In the first case, there are two consecutive waves propagating with different speeds (two-wave front or system of waves).

### Convergence to waves and systems of waves

In this section we address the question of how the travelling wave- or system of waves solutions of Eq (3) evolve from the specific initial conditions. Behavior of solutions of Eq (3) is described by travelling waves or by systems of waves if the waves do not exist [69]. We consider Eq (3) on the whole axis with the initial condition *v*(*x*, 0) = *v*_0_(*x*), where *v*_0_(*x*) is a step monotone function, *v*_0_(*x*) ≡ 0 for *x* ≥ 0 and *v*_0_(*x*) ≡ v_*_ > 0 for *x* < 0. Although much more general initial conditions can be considered, we restrict ourselves here to this particular case for simplicity of presentation.

For the initial values specified above the following conditions determine the evolution of solutions.

#### Convergence to the wave with the minimal speed

If 0 < v_*_ < *v*_2_, then solution of Eq (3) with initial condition *v*(*x*, 0) = *v*_0_(*x*) converges to the travelling wave with the minimal speed as follows:

$$\underset{x\in R}{\sup}|v\left(x+m_{0}\left(t\right),t\right)-w_{0}\left(x\right))|\to 0,\;\;\;t\to \infty ,$$ (10)

where $m_{0}^{\prime }\left(t\right)\to c_{0}$, *w*_0_(*x*) is a solution of Eq (5) with *c* = *c*_0_ and limits Eq (7) at infinity. The convergence holds both for the shape and speed of the solution pattern.

If v_*_ > *v*_2_ and *c*_1_ > *c*_0_ (the waves with the limits Eq (9) exist), then the solution also converges to the wave with the minimal speed c_*_. Note that behavior of solutions is different if v_*_ = *v*_2_. However this case is not a generic one and we will not consider it here.

#### Convergence to systems of waves

If *c*_1_ ≤ *c*_0_ and v_*_ > *v*_2_, then solution *v*(*x*, *t*) converges to the system of two waves propagating one after another with different speeds. This means that there are two functions *m*_1_(*t*) and *m*_2_(*t*) such that

$$\underset{x\in R_{+}}{\sup}|v\left(x+m_{0}\left(t\right),t\right)-w_{0}\left(x\right))|\to 0,\;\;\;t\to \infty ,$$ (11)

$$\underset{x\in R_{-}}{\sup}|v\left(x+m_{1}\left(t\right),t\right)-w_{1}\left(x\right))|\to 0,\;\;\;t\to \infty ,$$ (12)

where *w*_0_(*x*) is a solution of Eq (5) with *c* = *c*_0_ and limits Eq (7), *w*_1_(*x*) is a solution of Eq (5) with *c* = *c*_1_ and limits Eq (8), $m_{0}^{\prime }\left(t\right)\to c_{0}$, $m_{1}^{\prime }\left(t\right)\to c_{1}$, $R_{+}=\left\{x\geq x_{+}\right\}$, *R*_−_ = {*x* ≤ *x*_−_}. Here *x*_+_ and *x*_−_ are arbitrary real numbers, such that convergence Eq (11) occurs on an arbitrary (but fixed) right-half axis, and convergence Eq (12) on an arbitrary left-half axis.

### Determination of wave speeds

As it is indicated in the above section, existence of waves and behavior of solutions of Eq (3) depend on the values *c*_0_ and *c*_1_ of wave speeds. In this section we derive parameterized estimates of the wave speeds.

To find *c*_0_, consider the function *F*(*w*) on [0, *v*_1_]. The following inequality is true:

$$F^{\prime }\left(w\right)\leq F^{\prime }\left(0\right),\;\;\;0\leq w\leq v_{1}$$ (13)

since *f*′(*w*) ≥ 0 on this interval. Therefore, the following expression is valid for $c_{0}=2\sqrt{DF^{\prime }\left(0\right)}=2\sqrt{1-f(0)}$ (we recall that *D* = 1, *k* = 1).

Next, we derive the equation for estimation of *c*_1_. It admits an analytical formula for some particular forms of the function *F*(*w*), and there is a minimax representation of the wave speed for more general functions [69]. We aim to specify a simple condition for positivity of the wave speed and an analytical approximation for its value.

Multiplying Eq (5) by *w*′ and integrating, we obtain

$$c=\int_{v_{1}}^{v_{3}}F\left(w\right)dw/\int_{-\infty }^{\infty }{\left(w^{\prime }\left(x\right)\right)}^{2}dx\;.$$ (14)

This formula does not allow us to find the wave speed since *w*′(*x*) is unknown. However we can determine the sign of the wave speed. It is positive (zero, negative) if and only if the integral in the numerator is positive (zero, negative). Positiveness of the wave speed is important from the biological point of view because it signifies that infection propagates in the tissue. If it is negative, infection does not propagate.

Set $I\left(F\right)=\int_{v_{1}}^{v_{3}}F\left(w\right)dw$. Then the positivity condition *I*(*F*)>0 is equivalent to the condition

$$\int_{v_{1}}^{v_{3}}vf\left(v\right)dv<\int_{v_{1}}^{v_{3}}v\left(1-v\right)dv=\frac{1}{2}\left(v_{3}^{2}-v_{1}^{2}\right)-\frac{1}{3}\left(v_{3}^{3}-v_{1}^{3}\right).$$ (15)

Let us further approximate the function *F*(*w*) by a piece-wise linear function:

$$F\left(w\right)=\left\{\begin{array}{ccc} a(v_{1}-w) & , & v_{1}\leq w\leq w_{*}\\ b(v_{3}-w) & , & w_{*}<w\leq v_{3} \end{array}\right.\;,$$ (16)

where *a* and *b* are some positive constants. Since solution of Eq (5) is invariant with respect to translation in space, then we can set *w*(0) = w_*_ and write this equation for *x* > 0 and *x* < 0 as follows:

$$\begin{array}{ccc} w^{\prime \prime }+cw^{\prime }+a\left(v_{1}-w\right)=0 & , & x>0\\ w^{\prime \prime }+cw^{\prime }+b\left(v_{3}-w\right)=0 & , & x<0 \end{array}\;.$$ (17)

Therefore we find:

$$w\left(x\right)=v_{1}+\left(w_{*}-v_{1}\right)e^{-\lambda x}\;,\;\;x\geq 0;\;\;\;w\left(x\right)=v_{3}-\left(v_{3}-w_{*}\right)e^{\mu x}\;,\;\;x\leq 0,$$ (18)

where

$$\lambda =\frac{c}{2}+\sqrt{\frac{c^{2}}{4}+a}\;,\;\;\;\;\mu =-\frac{c}{2}+\sqrt{\frac{c^{2}}{4}+b}\;.$$ (19)

From the condition that the first derivative of the solution is continuous at *x* = 0, we obtain the following equation with respect to *c*:

$$\lambda \left(w_{*}-v_{1}\right)=\mu \left(v_{3}-w_{*}\right)$$ (20)

or

$$\phi \left(c\right)=b\;\frac{v_{3}-w_{*}}{w_{*}-v_{1}}\;,\;\;\;\text{where}\;\;\phi \left(c\right)=\left(\frac{c}{2}+\sqrt{\frac{c^{2}}{4}+a}\right)\left(\frac{c}{2}+\sqrt{\frac{c^{2}}{4}+b}\right)\;.$$ (21)

Let us recall that the large amplitude travelling waves with the limits Eq (9) exist if *c*_1_ > *c*_0_ and do not exist if *c*_1_ ≤ *c*_0_. Since *c*_1_ satisfies equality Eq (21) and $c_{0}=2\sqrt{1-f(0)}$, then the critical condition *c*_1_ = *c*_0_ for the travelling wave existence can now be written as follows:

$$\phi \left(c_{0}\right)=b\;\frac{v_{3}-w_{*}}{w_{*}-v_{1}}.$$ (22)

This equality represents a hypersurface in the model parameter space, i.e, *a*, *b*, w_*_, *v*_1_, *v*_3_, separating the regions of existence and non-existence of travelling waves with large amplitude. For all other parameters fixed, it determines the critical value *a*_0_ as a solution of this equation such that the waves exist for *a* < *a*_0_ and they do not exist for *a* ≥ *a*_0_.

### Regimes of spatiotemporal propagation

Under the simplifying assumption of no delay in the development of the immune response, which can be reasonable for slowly replicating viruses, the virus infection spreading is described by Eq (3). There are different spatiotemporal regimes of its propagation depending on the value v_*_, which can be interpreted as the initial virus load, and on the values *c*_0_ and *c*_1_ of the wave speeds, which in turn are determined by the model parameters. Depending on the initial dose of infection and on the intensity of immune response (function *f*(*v*)) the model predicts three different regimes of infection spreading (see also Fig 2).

#### Low dose infection

If v_*_ < *v*_2_ then infection spreads through the organ with the speed *c*_0_ and establishing the final level of viral load *v* = *v*_1_. Similar scenario holds for v_*_ > *v*_2_ and *I*(*F*)≤0 although the regime of propagation is different. In the first case, the infection front spreads resulting in the low level virus persistence in the organ. There is no transition from the low level to the high level infection with *v* > *v*_1_. In the second case, the low viral load infection front spreads, but there is a transition from the low level infection to the high level infection. This second front retreats (negative speed), and the region of low value infection expands in both directions.

#### High dose infection

If the initial viral infection load is high (v_*_ > *v*_2_) and the parameters of the model are such that *c*_1_ > *c*_0_, then infection front propagates establishing a persistent infection with a high level of viral load *v* = *v*_3_. Its speed of propagation is greater than the speed of the low viral load infection. The condition on the critical wave speeds can be approximated analytically (Section “Determination of wave speeds”).

#### Two step propagation

Finally, if the initial infection load is high (v_*_ > *v*_2_), *c*_1_ ≤ *c*_0_ and *I*(*F*) > 0, then there are two consecutive infection fronts. First, there is a front with low infection level behind it. This first front is followed by transition from the low level infection to the high level infection. It propagates with a lesser speed than the first one.

Let us note that increase of the parameter *a* (which corresponds to the increase of immune response) leads to the transition from the regime with high infection value to the regime with two step propagation and then to the regime with low infection value.

## Model problems with time delay

A qualitative analysis of the behaviour of solutions of the delay reaction-diffusion model for a general bell-shaped immune response function *f*(⋅) presents a formidable challenge. We proceed using simplifying approximations to it.

In this section we consider Eq (3) with a piece-wise constant function *f*(*v*):

$$f\left(v\right)=\left\{\begin{array}{ccc} a & , & v\leq w_{0}\\ b & , & v>w_{0} \end{array}\right.,$$ (23)

where *a*, *b* ≥ 0 and *w*_0_ > 0 are some constants. If *a* < *b*, then it is an approximation of the growing branch of the function *f*(*v*) in Fig 1, if *a* > *b*, of its decreasing branch. We look for conditions of the existence of a travelling wave solution *v*(*x*, *t*) = *w*(*x* − *ct*), which satisfies the equation

$$w^{\prime \prime }\left(x\right)+cw^{\prime }\left(x\right)+w\left(x\right)\left(1-w(x)-f(w(x+c\tau ))\right)=0$$ (24)

with the following boundary conditions

w(-∞)=1-b,w(+∞)=0. (25)

We further assume that *w*_0_ < 1 − *b*. We look for a solution of problem Eqs (24) and (25). Since it is invariant with respect to translation in space, we suppose that the discontinuity of the function *f*(*w*(*x* + *cτ*)) occurs for *x* = 0, that is *w*(*cτ*) = *w*_0_. Hence instead of Eq (23) we can write

$$\left\{\begin{array}{ccc} w^{\prime \prime }\left(x\right)+cw^{\prime }\left(x\right)+w\left(x\right)\left(1-w\left(x\right)-b\right)=0 & , & x\leq 0\\ w^{\prime \prime }\left(x\right)+cw^{\prime }\left(x\right)+w\left(x\right)\left(1-w\left(x\right)-a\right)=0 & , & x>0 \end{array}\right..$$ (26)

These equations should be completed by the relations

$$w\left(-0\right)=w\left(+0\right),\;\;\;w^{\prime }\left(-0\right)=w^{\prime }\left(+0\right),\;\;\;w\left(c\tau \right)=w_{0}$$ (27)

and by condition (25). Since eq (26) do not contain delay, we will use the phase space analysis to find the trajectories which satisfy condition (27).

### Bistable case

We begin with the case where 0 < *b* < 1 < *a* in which the piece-wise constant function (23) approximates decreasing branch of the function *f*(*w*) in Fig 1 where immune response decreases with the increase of infection level. Under these assumptions the stationary points *w* = 0 and *w* = 1 − *b* of the corresponding equation *dw*/*dt* = *F*(*w*) are stable. Instead of the second-order equations in Eq (26) we will consider two systems of the first-order equations:

$$w^{\prime }=p,\;\;\;p^{\prime }=-cp-kw\left(1-b-w\right)\;;\;\;\;\;w^{\prime }=p,\;\;\;p^{\prime }=-cp-kw\left(1-a-w\right).$$ (28)

Analysis of the trajectories of these systems allows one to formulate the following statement.

**Proposition 1.** *Let a* > 1, 0 < *w*_0_ < 1 − *b and*

$$3\left(a-b\right)w_{0}^{2}<{\left(1-b\right)}^{3}$$ (29)

*Then for any τ > 0 there is a unique positive value of c for which there exists a solution of problem* Eqs (25)–(27).

The proof of this statement is given in (S1 Appendix). Condition (29) ensures the positiveness of the wave speed *c*. It can be shown that the wave speed decreases if *τ* increases. To this end we consider the following approximate model obtained via linearization of system (26) about the boundary values at infinity:

$$\left\{\begin{array}{ccc} w^{\prime \prime }\left(x\right)+cw^{\prime }\left(x\right)+\left(1-b\right)\left(1-w\left(x\right)-b\right)=0 & , & x\leq 0\\ w^{\prime \prime }\left(x\right)+cw^{\prime }\left(x\right)+\left(1-a\right)w\left(x\right)=0 & , & x>0 \end{array}\right..$$ (30)

This approximating system admits an analytical solution and allows one to find the speed of infection wave propagation as a function of the model parameters. We have from Eq (30):

$$1-b-w\left(x\right)=k_{1}e^{\lambda x}\;,\;\;\;x\leq 0;\;\;\;\;w\left(x\right)=k_{2}e^{-\mu x}\;,\;\;\;x>0,$$ (31)

where

$$\lambda =-\frac{c}{2}+\sqrt{\frac{c^{2}}{4}+1-b}\;,\;\;\;\mu =\frac{c}{2}+\sqrt{\frac{c^{2}}{4}+a-1}\;.$$ (32)

From condition (27) we get the following equations:

$$1-b-k_{1}=k_{2},\;\;k_{1}\lambda =k_{2}\mu ,\;\;\;k_{2}e^{-\mu c\tau }=w_{0}.$$ (33)

From these equations we obtain the following relationship between the wave speed and time delay in immune response. We can write it as an explicit analytical dependence of *τ* on *c*:

$$\tau =\frac{1}{\mu c}\;\log\frac{{\left(1-b\right)}^{3/2}}{w_{0}\left(\sqrt{\frac{c^{2}}{4}+1-b}+\sqrt{\frac{c^{2}}{4}+a-1}\right)\left(\frac{c}{2}+\sqrt{\frac{c^{2}}{4}+1-b}\right)}\;.$$ (34)

**Fig 3:**
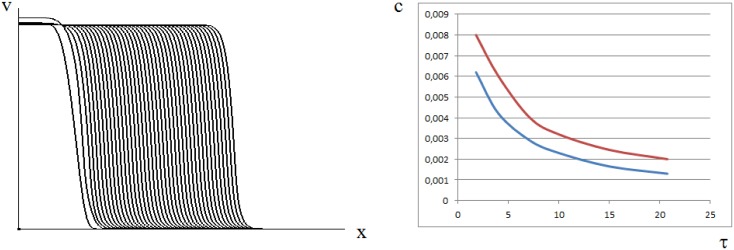
Wave propagation in the bistable case (left) for Eq (1) with function *f*(*v*) given by formula (23) (*a* = 1.1, *b* = 0.1, *w*_0_ = 0.1, *D* = 10^−4^). The curves show the function *v*(*x*, *t*) at successive moments of time. The wave speed dependence on time delay (right). The lower curve shows the results of numerical simulations, the upper curve is the analytical approximation by formula (34).

Fig 3 shows an example of wave propagation and comparison of the analytical and numerical results for the wave speed (see S2 Appendix for details). The wave speed decreases with time delay. From the biological point of view, this means that infection wave propagates slower if the delay in the clonal expansion of the antiviral immune response gets longer.

### Monostable case

As above, we consider problem Eqs (24) and (25) with function (23) and reduce it to problem Eqs (25)–(27). We will assume in this section that 0 < *a* < *b* < 1 and 0 < *w*_0_ < 1 − *b*. Then the first system in Eq (28) has two stationary points for 0 ≤ *w* ≤ 1, (0, 0) is a a node or a focus, depending on the value of *c*, and (1 − *b*, 0) is a saddle point. The second system in Eq (28) has only one stationary point (0, 0), which is a node or focus. We suppose that $c\geq 2\sqrt{1-a}$. Then the point (0, 0) is a stable node for both systems.

**Proposition 2.** *Suppose that 0 < a < b < 1 and 0 < w_0_ < 1 − b. Then for any* $c\geq 2\sqrt{1-a}$ *and τ > 0 there exists a solution of problem* Eqs (24) and (25) *with* function (23).

The proof of this proposition is given in (S1 Appendix). It can be verified that the solution becomes non-monotone for *τ* sufficiently large.

We now provide an analytical approximation to the solution of the system. Solution of eq (30) changes if *a* < 1 since there are two bounded exponentials for *x* > 0:

$$1-b-w\left(x\right)=k_{1}e^{\lambda x}\;,\;\;\;x\leq 0;\;\;\;\;w\left(x\right)=k_{2}e^{-\mu_{1}x}+k_{3}e^{-\mu_{2}x}\;,\;\;\;x>0,$$ (35)

where

$$\lambda =-\frac{c}{2}+\sqrt{\frac{c^{2}}{4}+1-b}\;,\;\;\;\mu_{1,2}=\frac{c}{2}\pm \sqrt{\frac{c^{2}}{4}+a-1}\;,$$ (36)

Representation of solution Eq (35) holds if *μ*_1_ ≠ *μ*_2_. From condition (27) we get the following equations:

$$1-b-k_{1}=k_{2}+k_{3},\;\;k_{1}\lambda =k_{2}\mu_{1}+k_{3}\mu_{2},\;\;\;k_{2}e^{-\mu_{1}c\tau }+k_{3}e^{-\mu_{2}c\tau }=w_{0}.$$ (37)

Expressing *k*_1_ from the first equation, we obtain the system of two equations with respect to *k*_2_ and *k*_3_:

$$\left(\mu_{1}+\lambda \right)k_{2}+\left(\mu_{2}+\lambda \right)k_{3}=\left(1-b\right)\lambda ,\;\;\;k_{2}e^{-\mu_{1}c\tau }+k_{3}e^{-\mu_{2}c\tau }=w_{0}.$$ (38)

Since *μ*_1_ > *μ*_2_ > 0, λ > 0, *τ* > 0, then, assuming that *c* > 0, we have

$$\left(\mu_{1}+\lambda \right)e^{-\mu_{2}c\tau }>\left(\mu_{2}+\lambda \right)e^{-\mu_{1}c\tau }\;.$$ (39)

Therefore the determinant of system (38) is positive, and it has unique values of *k*_2_, *k*_3_ for any *c* and *τ*.

We obtain below an analytical representation of the approximate solution for the minimal speed *c*_0_. Instead of Eq (35) we now have:

$$1-b-w\left(x\right)=k_{1}e^{\lambda x}\;,\;\;\;x\leq 0;\;\;\;\;w\left(x\right)=k_{2}e^{-\mu x}+k_{3}xe^{-\mu x}\;,\;\;\;x>0,$$ (40)

where *μ* = *c*/2, and from Eq (27) it follows that

$$1-b-k_{1}=k_{2},\;\;k_{1}\lambda =k_{2}\mu -k_{3},\;\;\;\left(k_{2}+k_{3}c\tau \right)e^{-\mu c\tau }=w_{0}.$$ (41)

**Fig 4:**
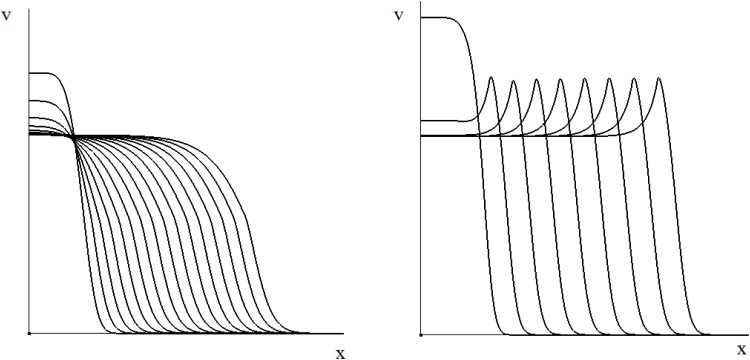
Propagating wave in the monostable case is monotone for small time delay (*τ* = 1, left) and non-monotone for large time delay (*τ* = 8, right). The values of other parameters are *a* = 0.1, *b* = 0.3, *w*_0_ = 0.1, *D* = 10^−4^.

Hence we get

$$k_{1}=1-b-k_{2},\;\;\;k_{2}=\frac{w_{0}e^{\mu c\tau }+\left(1-b\right)\lambda c\tau }{1+(\lambda +\mu )c\tau }\;,$$ (42)

which suggest that the solution is increasing for *x* < 0 if *k*_1_ < 0. Therefore the travelling wave is non-monotone for *τ* sufficiently large (Fig 4).

Solution *w*(*x*) is positive and monotonically decreasing for *x* > 0 if *μ*_1_ and *μ*_2_ are real and positive. This condition is satisfied for $c\geq 2\sqrt{1-a}$. Hence we consider the values of speed greater than or equal to the minimal speed $c_{0}=2\sqrt{1-a}$. Numerical simulations with the reaction-diffusion model presented in Fig 4 show that, similar to the case without delay, solution *v*(*x*, *t*) with the initial condition *v*(*x*, 0) such that *v*(*x*, 0) ≥ 0 for all *x*, *v*(*x*, 0)¬ ≡ 0 and *v*(*x*, 0)≡0 for *x* sufficiently large, approaches the travelling wave with the minimal speed. The details of the numerical solution of the delay reaction-diffusion model are given in S2 Appendix.

### Time oscillations

In this section we analyse the emergence of travelling wave solutions with a periodic structure established after the wave propagation. We do not assume here that the function *f*(*v*) is given by equality Eq (23). Let the following equality hold *f*(*v*_0_) = 1 − *v*_0_ for some *v*_0_, and *f*′(*v*_0_) > 0. Then *v*(*x*, *t*) = *v*_0_, *x* ∈ (−∞, +∞), *t* ∈ (−∞, +∞) is a stationary solution of Eq (1). In order to study its local asymptotic stability with respect to small perturbations, we look for the solution of this equation in the form

$$v\left(x,t\right)=v_{0}+\epsilon e^{\lambda t+iax}\;,$$ (43)

where *a*, *ϵ* are real numbers, *ϵ* is a small parameter, and λ is an eigenvalue. Substituting the above function in Eq (1) and equating the terms with the first power of *ϵ*, we get the following characteristic equation:

$$\lambda =-Da^{2}-v_{0}\left(1+f^{\prime }\left(v_{0}\right)e^{-\lambda \tau }\right)$$ (44)

(we do not assume here that *D* = 1). The stability boundary of the steady state solution can be computed by considering the characteristic roots in the form of purely imaginary eigenvalues λ = *iϕ*. Then we have

$$i\phi =-Da^{2}-v_{0}\left(1+f^{\prime }\left(v_{0}\right)e^{-i\phi \tau }\right).$$ (45)

Therefore the following equalities must hold for the real and imaginary parts:

$$Da^{2}+v_{0}+v_{0}f^{\prime }\left(v_{0}\right)\cos\left(\phi \tau \right)=0,$$ (46)

$$v_{0}f^{\prime }\left(v_{0}\right)\sin\left(\phi \tau \right)=\phi .$$ (47)

Set *z* = *ϕτ*. Then from Eqs (46) and (47) we obtain

$$\cos z=-\frac{Da^{2}+v_{0}}{v_{0}f^{\prime }\left(v_{0}\right)}\;,\;\;\;\tau =\frac{z}{\left(v_{0}f^{\prime }\left(v_{0}\right)\sin z\right)}\;.$$ (48)

The first equation has a solution if the right-hand side is greater than −1. In particular, if *D* = 0, then the condition reduces to *f*′(*v*_0_) ≥ 1. Using *z* determined from the first equation, we find *τ* from the second equation.

**Proposition 3.** *If f′*(*v*_0_) > 1 + *Da*^2^/*v*_0_, *then for all τ* > *z*/(*v*_0_ *f*′(*v*_0_)sin *z*) *the solution v*_0_ *of* Eq (1) *is unstable. Here z is determined from the first equation in* Eq (48).

Our analysis suggests that if the initial condition *v*(*x*, 0) does not depend on the space variable, then the solution *v*(*x*, *t*) of Eq (1) is also homogeneous in space. In this case, depending on the values of parameters, the solution of the model either convergence to the stationary solution *v*_0_ or to stable periodic time oscillations both being spatially homogeneous.

However, this behavior can be different in the case of travelling wave propagation. If we fulfil the linear stability analysis of the homogeneous in space stationary solution *v*_0_ in the moving coordinate frame attached to the wave, we obtain the same stability conditions as before. It follows from the first equation in Eq (48) that the onset of stability depends on the wave number *a* of the spatial perturbation and on the diffusion coefficient. The steady-state solution *v*_0_ appears to be more stable with respect to spatially non-uniform perturbations (*a* ≠ 0) than with respect to perturbations which are homogeneous in space (*a* = 0). The frequency of the spatial perturbations depends on the ratio of wave speed and the frequency of the time oscillations, *a* = *ϕ*/*c*.

**Fig 5:**
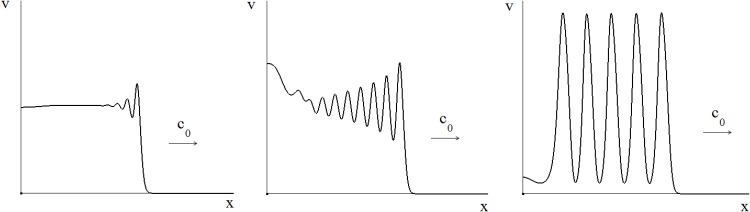
Numerical simulations of eq (1) with the function *f*(*v*) = *rv* (*r* = 2, *D* = 10^−4^). Wave propagation for three different values of time delay, *τ* = 1.4, 2, 4, respectively. For small time delay (left) space and time oscillations decay, for intermediate time delay (middle) space oscillations decay while time oscillations persists, for large time delay (right) both of them persist.

Hence the model predicts the existence of three spatiotemporal regimes of travelling wave propagation summarized in Fig 5 in the case of the linear function *f*(*v*) = *rv*. In the first one, both types of perturbations, i.e., the spatial perturbations and time perturbations homogeneous in space, decay with time. They manifest themselves as decaying spatial oscillations behind the wave front followed by a spatially constant solution (Fig 5, left). Another regime of wave propagation is characterized by the decaying spatially heterogeneous perturbation and the persisting homogeneous in space perturbations (Fig 5, middle).

Finally, if the spatial perturbations persist, then the travelling wave propagation takes place with a (moving) periodic structure established behind the wave front (Fig 5, right). Note that the wave speed in all these cases equals *c*_0_.

## Full-scale viral regulation of immune response

We proceed with the analysis of the spatiotemporal pattern formation for model 1. In the previous section we studied analytically and numerically three distinct reduced complexity models that correspond to various specific modes of immune response regulation, i.e. activation, suppression. The first one models the virus-driven activation of the immune response with a growing function *f*(*v*), the second variant corresponds to the down-regulation of immune response with a decreasing function *f*(*v*). In both of the above cases we considered step functions in order to get some analytical results about the generic behavior of solutions. The third model problem considers a growing function *f*(*v*) similar to the first version but with a continuous function for which we analyzed emergence of time oscillations. We consider now a full-scale viral regulation of the immune response described by Eq (1), i.e. the whole function *f*(*v*), *v* ≥ 0 as shown in Fig 1. The corresponding behavior of solutions will be assessed using numerical numerical simulations and insight gained via the analyses of the simplified model problems.

Let us recall that equation *f*(*v*) = 1 − *v* has three roots (solutions), *v*_1_, *v*_2_ and *v*_3_. In the model without delay (Section “Virus spread without time delay”), Eq (3) has wave solutions with the limits *w*(−∞) = *v*_1_, *w*(∞) = 0 for all values of the speed greater than or equal to the minimal speed $c_{0}=2\sqrt{D(1-f(0))}$. Similar result holds for the model problem in the monostable case (Section “Model problems with time delay”) when the delay *τ* > 0. Suppose that the stationary point *v*_1_ is stable (cf. Section “Time oscillations”), and the initial condition has limits 0 and *v*_1_ at ±∞. Numerical simulations show that solution of Eq (1) converges to the wave with the minimal speed. If the time delay is sufficiently large, then the wave is not monotone, similar to the model problems considered in Sections “Monostable case” and “Time oscillations”.

The travelling wave with the limits *w*(−∞) = *v*_3_, *w*(∞) = *v*_1_ corresponds to the bistable case. It exists for a unique value *c*_1_ of wavefront speed in the case without delay *τ* = 0 and for the model problem with non-zero delay *τ* > 0.

**Fig 6:**
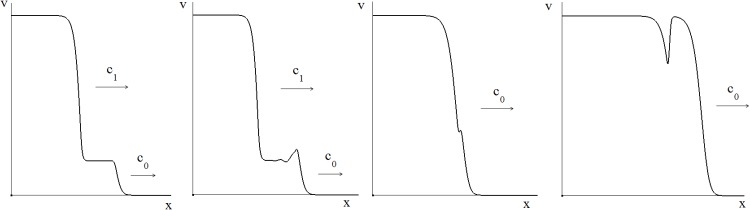
Numerical simulations of different regimes of infection spreading depending on time delay, *τ* = 0.4, 0.95, 1.5, 10;*D* = 0.0001. For small time delay (two left figures: *τ* = 0.4, 0.95), there are two consecutive waves of infection propagating with different speeds. The first wave can be non-monotone. For large time delay (two right figures: *τ* = 1.5, 10), the second wave propagates faster and they finally merge forming a single wave which can be either monotone or non-monotone.

Let us consider the case where *c*_1_ < *c*_0_. Then in the case without delay (*τ* = 0) the wave with the limits *w*(−∞) = *v*_3_, *w*(∞) = 0 does not exist. The behavior of solutions of Eq (1) is described by a system of two waves propagating one after another with the speeds *c*_0_ and *c*_1_. A similar behavior is observed when the delay is present in the regulation of the immune response but is sufficiently small (Fig 6). The solution can be monotone behind the second wave or non-monotone at all depending on the value of *τ*.

Contrary to the case considered in Section “Bistable case”, the speed of the bistable traveling wave increases with time delay. This increase occurs because the bistable wave is preceded by the a monostable wave characterized by a lower level of infection. Therefore the values of the function *f*(*v*) at the bistable wave also decrease due to time delay. Hence the speed *c*_1_ increases as a function of *τ*, while the speed *c*_0_ of the monostable wave does not depend on *τ*. For *τ* sufficiently large the two waves merge and form a single wave with the limits *w*(−∞) = *v*_3_, *w*(∞) = 0 (Fig 6). This effect does not exist in the model problems considered in Section “Model problems with time delay”. It is specific for the system of waves where the bistable wave follows the monostable one. If *τ* is large enough, this resulting wave becomes non-monotone.

**Fig 7:**
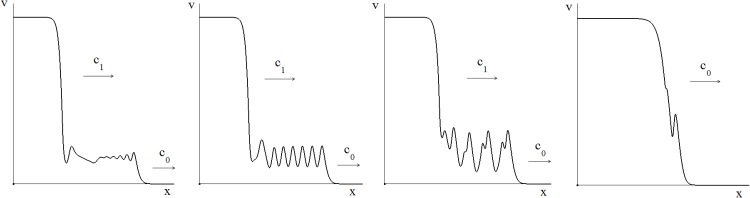
Spatiotemporal regimes of infection spreading. The monostable wave becomes non-monotone with decaying or persisting oscillations behind it. The type of patterns on the left is characterized by a transition zone between decaying space oscillations and the bistable wave with perturbed time oscillations of the homogeneous solution *v*_1_. Space oscillations become more complex for larger values of *τ* which represent a second type of the spatial dynamics. If time delay is sufficiently large, then the two travelling waves merge, as before, forming a single stable non-monotone wave. The values of time delay are, respectively, *τ* = 0.7, 1, 1.5, 2;*D* = 0.0001.

If *f*′(*v*_1_) is sufficiently large, i.e., the sensitivity of the T cell activation to the antigen is high then the stationary point *v*_1_ becomes unstable (Section “Time oscillations”). The spatiotemporal regimes of infection spreading show a broad spectrum of dynamic patterns. The monostable wave becomes non-monotone with decaying or persisting oscillations behind it (Fig 7). One type of patterns is characterized by a transition zone between decaying space oscillations and the bistable wave with perturbed time oscillations of the homogeneous solution *v*_1_. Space oscillations become more complex for larger values of *τ* which represent a second type of the spatial dynamics. If time delay is sufficiently large, then the two travelling waves merge, as before, forming a single stable non-monotone wave.

**Fig 8:**
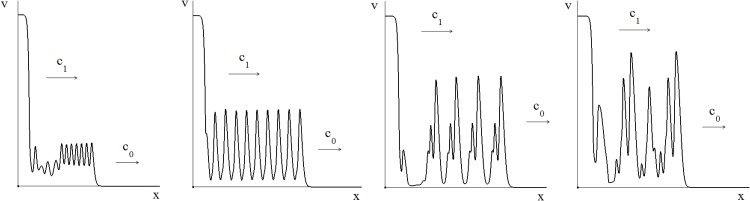
Existence of a monostable wave with spatial oscillations behind it. This wave is separated from the bistable wave by a zone of irregular oscillations. Increase of the delay value results in a qualitative change of the spatial patterns of the infection spread. The two travelling waves do not merge and the monostable wave is not followed by steady space oscillations. Aperiodic oscillations are observed behind the wave front which propagates at a speed *c*_0_. The values of time delay are, respectively, *τ* = 1, 2, 3, 4;*D* = 0.0001.

Fig 8 shows the last series of simulations in which the slope (sensitivity) of *f*′(*v*_1_) is larger than in the above set of simulations and the instability of the steady state solution *v*_1_ is more pronounced. In this case, we observe the existence of a monostable wave with spatial oscillations behind it. This wave can be separated from the bistable wave by a zone of irregular oscillations. Further increase of the delay results in a qualitative change of the spatial patterns of the infection spread. The two travelling waves do not merge any longer and the monostable wave is not followed by steady space oscillations. Aperiodic oscillations are observed behind the wave front which propagates, as before, at a constant speed *c*_0_.

**Fig 9:**
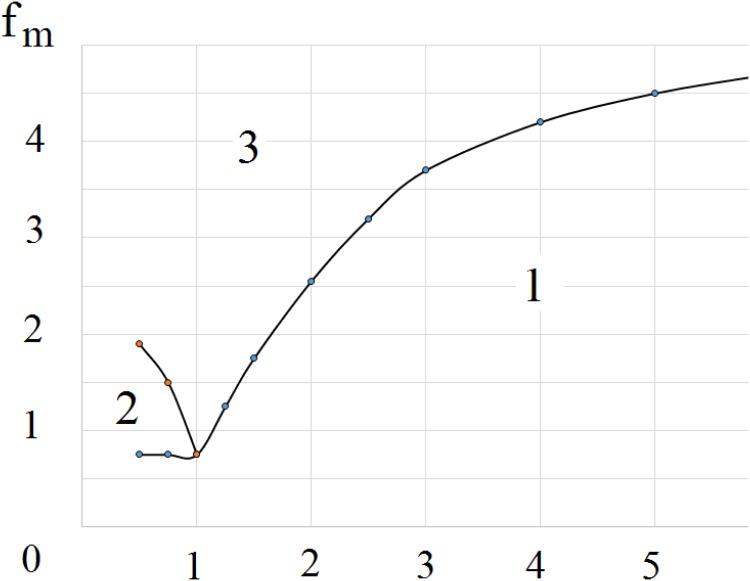
Different regimes of infection wave propagation on the parameter plane (*f*_*m*_ is the maximum of the function *f*(*v*), and *τ* is the time needed for the development of immune response), see Supplementary materials: 1—stationary wave propagation (cf. Fig 6, two right images, Fig 7, right image), 2—two consecutive waves with different speeds (cf. Fig 6, two left images), 3—two waves with spatiotemporal patterns between them (cf. Fig 7, three left images and Fig 8).

We summarize the analysis of the existence of various dynamical regimes of infection wave propagation on the parameter plane representing the strength of the immune response, characterized by parameter *f*_*m*_ and the time delay, representing the duration of the clonal expansion of the virus-specific T cells in Fig 9. For small delays and weak immune responses the infection spreads as a stationary wave (region 1, left low domain). An increase in the strength of the immune response leads to the emergence of two consecutive waves propagating with different speeds (region 2). Strong immune responses cause the infection spread in the form of two waves with spatiotemporal patterns between them for delays which are not too large (region 3). However, if the time needed for the immune response to develop exceeds a certain threshold (region 1) then the infection propagates, as before, as a stationary wave.

## Discussion

Viral propagation in host organisms during infection includes two processes, the local spreading inside permissive tissues and organs, and the global propagation between different organs. The target organs for HIV and SIV infections are lymphoid tissues such as GALT, lymph nodes, spleen, etc., whereas most other viruses replicate outside organized lymphoid organs, e.g., the hepatitis viruses (B and C) primarily infect and spread in the liver cells. In this study we suggest a novel mathematical model for the spatiotemporal dynamics of virus infection in the face of an antiviral immune response. The model is formulated with a 1D reaction-diffusion equation for the spatially distributed viral population. The reaction term considers the immune response regulated by a bell-shaped dose-response relationship with viral load. It takes into account the time-lag between the activation of the virus-specific T cells and the elimination of the virus by clonally expanded CTLs. The model was used to gain insights into plausible regimes of the viral spread in tissues. To this end a combination of analytical studies of the simplified versions of the model and numerical simulations were conducted to elucidate the role of key infection parameters such as dose, diffusion coefficient characterizing the efficacy of the cell-to-cell or free virus-to-cell spread, and delay in the establishment of the antiviral immune response.

Our mathematical model is based upon the following general phenomenology of virus infections. Viruses reproduce inside host cells and transport to new cells either by direct cell-cell contact or through the extracellular matrix. Infected cells are killed by CTLs. Thus, infection spreading in the tissue is determined by virus multiplication, virus transport and immune responses. The intensity of the immune response depends on the infection level. It has a specific bell shape as shown in Fig 1. The form of the function *f*(*v*) shows that the immune response intensifies with the infection level up to some critical value where this function attains its maximum. However, for greater virus levels, it decreases because viruses affect the cells that participate in immune response. Moreover, the immune response is not instantaneous, but requires some time for cell proliferation and maturation. Hence, in order to describe virus multiplication, transport and death we consider reaction-diffusion equations with a time delay. Further extensions of the model include the shift towards larger spatial dimensions, i.e, 2D- or 3D spatial models of viral spread and explicit consideration of the spatial-temporal dynamics of the immune responses in tissues [71, 72]. As the number of studies in the field is still rather limited as outlined in S1 Table, there is a number of challenges in terms of the modelling approaches and their computational treatment.

Although the proposed mathematical model is a one-dimensional non-linear PDE model with a time lag, the behavior of the model solutions is already quite complex. It depends on the intensity of immune response (function *f*(*v*)), the initial virus load (initial condition) and the growth rate of the virus in accordance with the existing view of the regulation of virus infections [1, 2, 4, 11, 29, 33, 34, 38]. We assume that the maximal intensity of the immune response is sufficiently high to overcome virus growth and spread in a low-dose infection. Under this assumption, the reaction-term equation *f*(*v*) = 1 − *v* has three positive solutions *v*_1_ < *v*_2_ < *v*_3_. The steady state solution *v*_1_ can be either asymptotically stable or unstable, i.e. exhibit an oscillatory regime, the steady state *v*_2_ is always unstable, *v*_3_ is always asymptotically stable.

The model predicts the existence of a diverse spectrum of spatiotemporal patterns of virus dynamics. The initial infection can convert the virus free steady-state *v* = 0 into one of the following infection states: a low level infection *v*_1_, a high level infection *v*_3_ or in oscillatory regime.

1. If the initial virus load is sufficiently low, then the model predicts the existence of a mono-stable wave propagating with the speed *c*_0_ determined by the diffusion coefficient *D* and by the immune response *f*′(0). The wave propagation depends on the value of the time delay *τ*. The wave front can be followed by either a constant low level infection *v*_1_ or a spatially periodic viral distribution moving through the tissue with the same speed *c*_0_.
2. If the initial virus load is sufficiently high, then the regimes of travelling wave propagation depend on the speed of the bistable wave *c*_1_. If it is negative, then the initial infection evolves to a low level spatially homogeneous infection. If this speed is positive but less than *c*_0_, then the infection spreads as a combination of two travelling waves. The first one, a low level travelling wave is followed by a transitory region of spatially heterogeneous or irregular infection finally replaced by a high level spatially homogeneous infection.
3. Finally, if *c*_1_ is sufficiently high, then there is only one travelling wave establishing a high infection level *v*_3_. This transition can be non-monotone as far as the viral distribution is concerned. Note that the speed of the bistable wave *c*_1_ increases with the increase of the time delay of the immune response.

The predicted patterns of virus infection spread admit the following biological interpretation. The traveling wave solution corresponds to an infection of the target organ spreading at some tempo resulting in a homogeneous or spatially periodic distribution of the virus in the organ. Depending on the parameters of the immune response and the virus diffusion, oscillatory dynamics can emerge indicating that the virus distribution in the organ can be spatially non-homogeneous and time-dependent. This could manifest as oscillations of the virus density with the amplitude different in different parts of the target organ. It should be noted, that oscillatory dynamics of the virus infection is considered to provide an evolutionary advantage to the virus by allowing it to evade the control by the immune response and to establish virus persistence [73]. In addition, the alternating phases of high- and low-virus replication levels allow the target cells to recover consumed resources and to circumvent cell death. Overall, the oscillatory dynamics seems to increase the robustness of virus persistence in face of the immune response. Virus distribution in lymph nodes can be highly inhomogeneous even if the tissue itself is homogeneous. A clear example is provided by HIV infection. Clinical and experimental studies of HIV and SIV infections indicate that the virus growth occurs in multiple local bursts reflecting local non-equilibrium interactions between HIV and immune activated cells [31]. It was further discussed in [32] that in chronic HIV infection efficient transmission of the virus is limited to microscopic clusters of T cell in lymphoid tissues suggesting that the continuity of virus production is a result of spatially separated bursts. Mathematically, this would correspond to an oscillatory or irregular spatially inhomogeneous distribution of virus revealed in our study.

Spatial aspects of virus replication and the impact of the immune response on virus dynamics were thoroughly studied for SIV infection of monkeys [10]. Viral replication in skin patches of monkeys was clearly shown to be spatially heterogeneous (Fig 2c and 2d) and dependent on the time needed for CTLs to reach the site of local virus replication (i.e., the delay between activation of antigen-specific CTLs and in situ infiltration). It was proposed that the spatial spreading of the virus underlies the dynamical escape from the immune response. A series of latest studies on HIV visualized in cerebellum and lymph node tissues [74, 75] showed different patterns of virus distribution. HIV infected cells were clustered in cerebellum but were dispersed in lymph node tissues. The studies conclude that a high variability in the amount of HIV in tissues within an individual deserves further experimental analysis with a special attention to precise location of tissue sampling as factor affecting the amount of HIV recovered (non-homogeneity). In vivo studies of virus distribution and spreading in target tissues are complemented by the analysis of the impact of virus infection parameters on the infection spread between cells in in vitro systems [76].

The above findings concerning the spatiotemporal dynamics of the virus spread have direct implications for experimental and clinical studies of the biology of infections. The invention of imaging and visualization technologies provided a novel type of spatial information on the infection-host interaction process [7, 11, 77–79]. To interpret the data and gain a quantitative understanding of the determinants of the infection process from the image-derived data, the application of mathematical models is needed [80]. Indeed, the spatial patterns of pathogen dynamics can be complex and far from being uniform [7], and require the invention of appropriate concepts, patterns and parameters for their analysis. The major work in this direction remains to be done. Our analysis suggests that viral spreading in tissues can proceed as a travelling wave (characterized by either a low- or a high viral load), spatially periodic or irregular oscillations, and a combination of those. The evidence for the existence of spatially heterogeneous patterns, called multifocal infection, of virus spread in HIV infection in lymphoid tissue has been published recently [7]. The images of the tissues with fluorescently labelled HIV RNA [7] allow a range of interpretations depending on the choice of the cross-section: a bistable travelling wave (see Fig 1A), monostable low-level infection Fig 1B, or oscillatory mode (Fig 1D).

The possibility that the infection spreads as waves differing in their amplitude suggests the existence of spatiotemporal mechanisms of disease pathogenesis. Indeed, due to a delay *τ* in the development of the immune response, the magnitude of the immune reaction at time *t* will be consistent with the level of stimulation at time *t* − *τ*. If the level of infection at time *t* is considerably different from that at the earlier time of the immune stimulation, then the magnitude of the response might appear to be out of consistency with the current level of infection *in situ*. This in turn would result either in a higher destruction of the infected tissues (which would be otherwise protected by exhaustion mechanisms) or in an insufficient response favoring virus persistence, depending on the kinetics. Therefore, the immune response and the infection kinetics should be highly coordinated to ensure a proper clearance of the infection.

If the virus is not eliminated from the infected host then a chronic or persistent virus infection is established. To diagnose chronic infections with respect to the presence and extent of a disease, and to assess the results of therapeutic interventions, the quantification of the infection process is necessary. A biopsy-based medical testing may be performed involving extraction of tissues for examining the distribution and intensity of the infectious process. A recent example of the study of viral reservoirs in HIV infection based on examination of lymph node and GALT biopsies from subjects under antiretroviral therapy is provided in [7]. It is of note that the sections represent 3 × 10^−4^% of the lymph node tissue and the pinch biopsy area is 3 × 10^−6^ *m*^2^ compared to the area of the intestine in an adult human being ∼300 *m*^2^. This enormous difference in scale rises the issue of how to ensure that the biopsy is informative if the viral infection spread can be spatially essentially heterogeneous as discussed above. Obviously this calls for the need to coordinate interdisciplinary studies of virologists and clinicians with mathematicians in order to clearly define in quantitative terms the spatial patterns of infection. Today infections in tissues are solely classified as diffuse, zonal, patchy, spotty, confluent, etc. Further insights into the virus infection spreading will require extension of the one-dimensional model to the two-dimensional consideration.

As “space” becomes a frontier in immunology [72, 77], further multidisciplinary research is needed towards a mechanistic explanation of these types of pathology signs on a firm basis of the virus ontogeny, the function of specific cells, mediators, and tissue responses analyzed from small blood and tissue samples using computational models with a final aim of identifying the right targets and design more efficient therapies [81].

## Supporting Information

S1 Appendix (PDF)

S2 Appendix (PDF)

S1 Table (PDF)
