# Supplementary material for: Spatiotemporal Dynamics of Virus Infection Spreading in Tissues
Source: PLoS One. 2016 Dec 20;11(12):e0168576. doi: 10.1371/journal.pone.0168576 (PMC5173377; doi:10.1371/journal.pone.0168576)
Supplement: S1 Appendix — (PDF) [file pone.0168576.s001.pdf]

Supporting information

S1 Appendix

Proof of wave existence

Bistable case

We begin with the case where $a > 1$. Then the stationary points $w = 0$ and $w = 1 - b$ of the corresponding equation $dw/dt = F(w)$ are stable. Instead of the second-order equations in (20) we will consider the systems of the first-order equations:

$$w' = p, \quad p' = -cp - kw(1 - b - w) \tag{1}$$

and

$$w' = p, \quad p' = -cp - kw(1 - a - w). \tag{2}$$

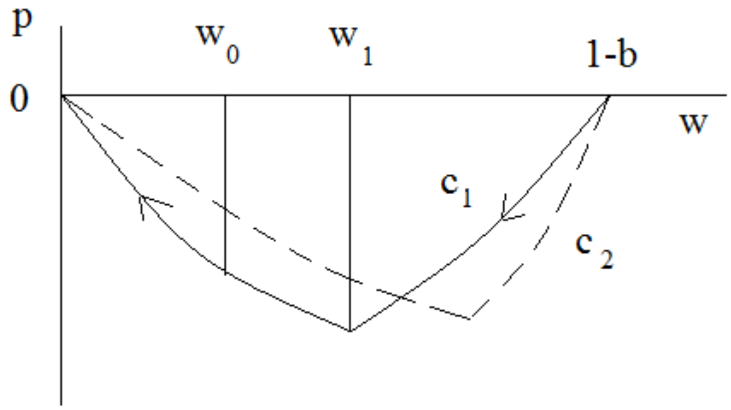

Fig 1. Trajectories of systems (1) and (2) for $c = c_1$ (solid lines) leaving the saddle point $(1 - b, 0)$ and approaching the saddle point $(0, 0)$. They intersect at $w = w_1$, that is $w(0) = w_1$ and $w(c\tau) = w_0$. The same trajectories for $c = c_2$ (dashed lines).

Consider the trajectory γ_1 of the first system for $c = c_1$ leaving the saddle point $(1 - b, 0)$ in the half-plane $p < 0$, and the trajectory γ_0 of the second system for $c = c_1$ approaching another saddle point $(0, 0)$ from the same half-plane.

Consider p as a function of w for the solutions of systems (1), (2), and denote by $p_1(w; c_1)$ the function corresponding to the trajectory γ_1. Similarly, let $p_0(w; c_1)$ be the function corresponding to the trajectory γ_0. Due to the properties of systems (1), (2), both these functions are defined on the interval $0 \le w \le 1 - b$, and

$$p_0(0; c_1) = 0, \quad p_0(1 - b; c_1) < 0; \quad p_1(0; c_1) \le 0, \quad p_1(1 - b; c_1) = 0.$$

Then there is a solution of the equation

$$p_0(w;c) = p_1(w;c) \tag{3}$$

$(c = c_1)$ for some $w_1 \in (0, 1-b)$. We have

$$\frac{dp_0(w_1;c_1)}{dw} = -c_1 - \frac{kw_1(1-a-w_1)}{p_0(w_1;c_1)} < -c_1 - \frac{kw_1(1-b-w_1)}{p_1(w_1;c_1)} = \frac{dp_1(w_1;c_1)}{dw}. \tag{4}$$

Therefore solution of Eq (3) in the interval $(0, 1-b)$ is unique.

Lemma 1. *Solution w_1 of Eq (3) is a decreasing function of c. Moreover, $w_1(c) = 0$ for $c \geq c_0$, and $w_1(c) \to 1-b$ as $c \to -\infty$. Here c_0 is the minimal speed for there exists a $[0, 1-b]$-trajectory of systems (1).*

Proof. It can be easily verified that

$$p_0(w;c_2) > p_0(w;c_1), \quad p_1(w;c_2) > p_1(w;c_1), \quad 0 < w < 1-b$$

for $c_2 < c_1$ (Fig 1). Therefore $w_1(c_2) > w_1(c_1)$.

Let us note that $(0,0)$ is a stable node of system (1) if $c \geq 2\sqrt{k(1-b)}$, and $(1-b, 0)$ is a saddle point. If the previous condition on c is satisfied, then there is a trajectory connecting them such that it entirely belongs to the half-strip $0 < w < 1-b$, $p < 0$. If $c < 2\sqrt{k(1-b)}$, then the trajectory connecting these stationary points exists, but it does not belong to the half-strip.

Thus, $p_1(0;c) = 0$ for $c \geq 2\sqrt{k(1-b)}$. Moreover $p_0(w;c) < p_1(w,c)$ for $0 < w \leq 1-b$. Indeed, since $p_0(0;c) = p_1(0;c) = 0$, then this assertion follows from the estimate (4) for the derivatives at the intersection point.

It remains to verify that $w_1(c) \to 1-b$ as $c \to -\infty$. It follows from the monotonicity of solutions with respect to c: the function $p_0(w;c)$ increases and the function $p_1(w;c)$ decreases for each w as c decreases.

By virtue of Lemma 1, there exists a unique value $c = c_0$ such that $w_1(c_0) = w_0$. The following lemma gives a condition under which $c_0 > 0$.

Lemma 2. *Let $w_1(c_0) = w_0$. Then $c_0 > 0$ if and only if*

$$3(a-b)w_0^2 < (1-b)^3. \tag{5}$$

Proof. It is sufficient to compare the values of the functions $p_0(w;c)$ and $p_1(w;c)$ for $w = w_0$ and $c = 0$. Namely, we require that $p_0(w_0;0) > p_1(w_0;0)$. From (1) we have for $c = 0$:

$$p\frac{dp}{dw} = -kw(1-b-w).$$

Integrating this equation between w_0 and $1-b$, we obtain

$$p_1^2(w_0;0) = 2k\left(\frac{1}{6}(1-b)^3 - \frac{1-b}{2}w_0^2 + \frac{1}{3}w_0^3\right).$$

Similarly, from (2),

$$p_0^2(w_0;0) = 2k\left(\frac{a-1}{2}w_0^2 + \frac{1}{3}w_0^3\right).$$

Condition (5) follows from the inequality $p_1^2(w_0;0) > p_0^2(w_0;0)$.

We can now prove the theorem on the existence of waves.

Theorem 3. *Let $a > 1$, $0 < w_0 < 1 - b$ and condition (5) be satisfied. Then for any $\tau > 0$ there is a unique positive value of c for which there exists a solution of problem (19)-(21).*

Proof. By virtue of Lemmas 1 and 2, there exists a unique value $c_0 > 0$ such that $w_1(c_0) = w_0$. Let us choose some c such that $w_1(c) > w_0$ and consider the second equation in (20). Then $w(0) = w_1(c)$ and $w(x_0) = w_0$ for some $x_0 > 0$. We will show that we can choose c such that $x_0 = c\tau$.

Let us determine how x_0 depends on c. Consider the trajectories of systems (1), (2) for c_1 and c_2 (Fig 1). If $c_2 < c_1$, then

$$p_0(w; c_1) < p_0(w; c_2), \quad w_0 \le w \le w_1(c_1). \tag{6}$$

Hence $x_0(c_2) > x_0(c_1)$. Indeed, the arc of the trajectory $p_0(w; c_2)$ consists of two parts. The first one between the values w_0 and $w_1(c_1)$, the second one between the values $w_1(c_1)$ and $w_1(c_2)$. Therefore we can set $x_0(c_2) = x_1(c_2) + x_2(c_2)$, where $x_1(c_2)$ and $x_2(c_2)$ are the lengths of the intervals corresponding to these arcs. It follows from inequality (6) that $x_1(c_2) > x_0(c_1)$. Then we get $x_0(c_2) > x_0(c_1)$.

Thus, $x_0(c_0) = 0$ and $x_0(c)$ is a decreasing function. Since $c_0 > 0$, then there exists a unique positive solution of the equation $x_0(c) = c\tau$.

Monostable case

As above, we consider problem (18), (19) with function (17) and reduce it to problem (19)-(21). We will assume in this section that $0 < a < b < 1$ and $0 < w_0 < 1 - b$. Then system (1) has two stationary points for $0 \le w \le 1$, $(0, 0)$ is a a node or a focus, depending on the value of c, and $(1 - b, 0)$ is a saddle point. System (2) has only one stationary point $(0, 0)$, which is a node or focus. We suppose that $c \ge 2\sqrt{(1-a)}$. Then the point $(0, 0)$ is a stable node for both systems.

Theorem 4. *Suppose that $0 < a < b < 1$ and $0 < w_0 < 1 - b$. Then for any $c \ge 2\sqrt{(1-a)}$ there exists a solution of problem (18), (19) with function (17).*

Proof. Consider the function $p_1(w; c)$ with $c \ge 2\sqrt{(1-a)}$ corresponding to the trajectory of system (1) leaving the stationary point $(1 - b, 0)$ into the half-plane $p < 0$. It approaches the point $(0, 0)$ since c is greater than the minimal wave speed $2\sqrt{(1-b)}$ for this system. Hence $p_1(0; c) = p_1(1 - b; c) = 0$.

Let $p_0(w; c)$ be the function corresponding to a trajectory of system (2) approaching the stationary point $(0, 0)$ and such that

$$p_0(w_1; c) = p_1(w_1; c) \tag{7}$$

for some $w_1 \in (0, 1 - b)$. Then

$$\frac{dp_0(w_1; c)}{dw} = -c - \frac{w(1 - a - w_1)}{p_0(w_1; c)} > -c - \frac{w(1 - b - w_1)}{p_1(w_1; c)} = \frac{dp_1(w_1; c)}{dw}. \tag{8}$$

This inequality determines the mutual direction of the trajectories of systems (1) and (2) at the intersection points (Fig 2).

Set

$$p(w) = \begin{cases} p_0(w; c) & , \quad 0 \le w \le w_1 \\ p_1(w; c) & , \quad w_1 \le w \le 1 - b \end{cases}$$

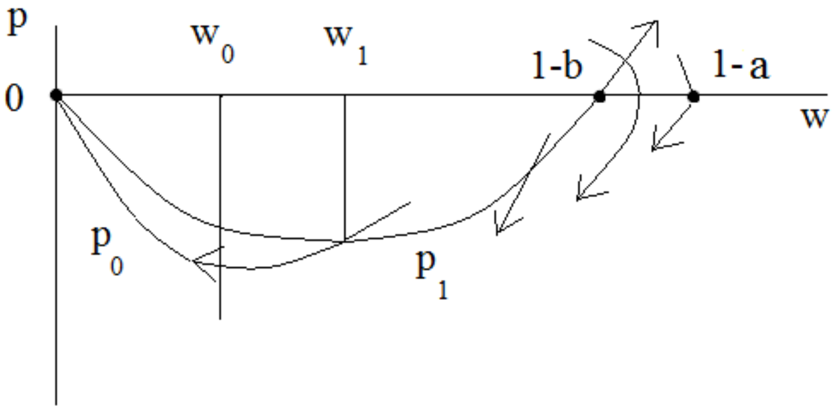

Fig 2. Trajectories of systems (1) and (2) in the monostable case.

and the corresponding function $w(x)$ such that $w'(x) = p(w(x))$. It is a solution of equations (20). It is continues together with its first derivative. We have $w(0) = w_1$, $w(x_0) = w_0$. We need to choose the value w_1 in order to satisfy the last condition in (21), that is $x_0 = c\tau$.

Let us note that for any $c \geq 2\sqrt{(1-a)}$, all trajectories of system (2) crossing the points $(w, p_1(w; c))$ on the trajectory of system (1) approach the stationary point $(0, 0)$. If we vary w_1 from w_0 to $1-b$, then the corresponding value of x_0 increases from 0 to some value which is finite since $(1-b, 0)$ is not a stationary point for system (2). Therefore there exists a maximal delay $\tau = \tau_m$ for which the solution remains monotone as a function of x. For $\tau > \tau_m$ we consider the intersection with the trajectory of system (1) leaving the stationary point $(0, 0)$ in the half-plane $p > 0$ (Fig 2). Therefore for any $\tau > 0$ there exists a value w_1 such that $x_0 = c\tau$. If $w_1 > 1-b$, then the solution is not monotone.
