# Supplementary material for: Spatiotemporal Dynamics of Virus Infection Spreading in Tissues
Source: PLoS One. 2016 Dec 20;11(12):e0168576. doi: 10.1371/journal.pone.0168576 (PMC5173377; doi:10.1371/journal.pone.0168576)
Supplement: S2 Appendix — (PDF) [file pone.0168576.s002.pdf]

Supporting information

S2 Appendix

Numerical method

Eq (1) is solved numerically with a finite difference approximation and an implicit scheme for the diffusion term:

$$\frac{v_i^{n+1} - v_i^n}{\delta t} = D\, \frac{v_{i+1}^{n+1} - 2v_i^{n+1} + v_{i-1}^{n+1}}{(\delta x)^2} \;+ v_i^n \left(1 - v_i^{n+1} - f(v_i^{n-k})\right). \tag{1}$$

Here the subscript i corresponds to the space discretization, n to time discretization, δx is the space step and δt is the time step. This equation is solved by the Thomas algorithm for the resolution of tridiagonal matrix equations.

Eq (1) contains the delay variable v_i^{n-k}, where k is determined by the equality $\tau = k(\delta t)$. For $n \leq k$ we set $v_i^{n-k} = v_i^0$, where v_i^0 is the discretized form of the initial condition. We use the Neumann (zero derivative) boundary conditions.

Using conventional approach of the resolution of delay equations, we need to keep in memory all functions $v_i^{n-k}, v_i^{n-k+1}, ..., v_i^n$. It becomes very resource consuming in the case of small time steps. With $10^3 - 10^4$ space discretization points used in the simulations and $\tau/\delta t$ up to 10^4 time discretization points, the required data can become excessively large.

We suggest here a different method to solve delay equations. Instead of Eq (1) consider the equations

$$\frac{\partial V_j}{\partial t} = D\, \frac{\partial^2 V_j}{\partial x^2} + V_j \left(1 - V_j - f(V_{j-1})\right), \quad j = 1, 2, 3, ..., t \geq (j-1)\tau, \tag{2}$$

where $V_j(x,t) = V^0(x)$ for $t \leq (j-1)\tau$ and $V^0(x)$ is the initial condition. Hence each function $V_j(x,t)$ satisfies the same problem with time shift, $V_j(x, t-\tau) = V_{j-1}(x,t)$. The discretization of these equations is similar to (1).

Thus, we replace one delay equation by a system of equations without delay whose number grows with time. If the total time interval is not very large, then this method is more efficient from the point of view of required memory. This difference is even more essential in the 2D case. The disadvantage of this method is that the equality $V_j(x, t-\tau) = V_{j-1}(x,t)$ should be verified exactly. This is obviously the case from the theoretical point of view because these are solutions of the same problem. This property should be carefully verified in the numerical code. Otherwise the error accumulation can result in completely wrong solutions.

Function $f(v)$

The function $f(v)$ used in numerical simulations is determined by the values $f(v_i)$, $i = 1, 2, 3, 4$ (Fig 1). The function is constant for $v \leq v_1, v_2 \leq v \leq v_3, v \geq v_4$. It is given by third-order polynomials in the intervals $v_1 \leq v \leq v_2$ and $v_3 \leq v \leq v_4$ constructed in such a way that the function is continuous together with its first derivatives.
For the function shown in Fig 1 we set $v_2 = v_3$. In Section "Bistable case" we put $v_1 = 0.1, v_2 = 1.01,\ f_1 = 1.1, f_2 = f_3 = f_4 = 0.1$ (the values v_3 and v_4 are not essential).

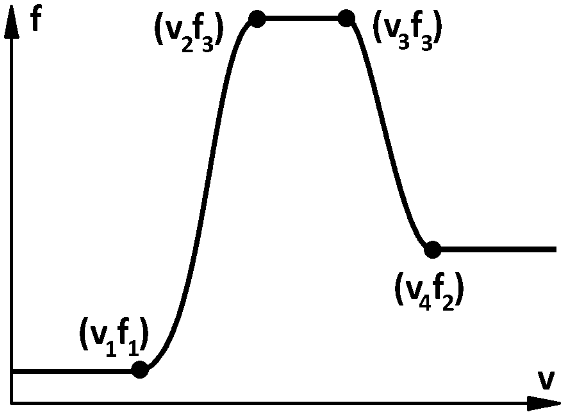

Fig 1. The structure of the function $f(v)$ used in numerical simulations.

In Section "Monostable case",

$v_1 = 0.1, v_2 = 1.01,\ f_1 = 0.1, f_2 = f_3 = f_4 = 0.3.$

Finally, in Section "Full-scale regulation of immune response" we consider three sets of parameter values:

$v_1 = 0.1, v_2 = v_3 = 0.2, v_4 = 0.3,\ f_1 = f_2 = 0.1, f_3 = 1$ (Fig 6);

$v_1 = 0.1, v_2 = v_3 = 0.2, v_4 = 0.3,\ f_1 = f_2 = 0.1, f_3 = 2$ (Fig 7);

$v_1 = 0.1, v_2 = v_3 - 0.3, v_4 = 0.5,\ f_1 = f_2 = 0.1, f_3 = 3$ (Fig 8);

$v1 = 0:1, v2 = v3 = 0:2, v4 = 0:3, f1 = f2 = 0:1, f_m = f_3$ (Fig 9).
