# Supplementary material for: Spatiotemporal Dynamics of Virus Infection Spreading in Tissues
Source: PLoS One. 2016 Dec 20;11(12):e0168576. doi: 10.1371/journal.pone.0168576 (PMC5173377; doi:10.1371/journal.pone.0168576)
Supplement: S1 Table — (PDF) [file pone.0168576.s003.pdf]

Supporting information

S1 Table

S1 Table. Overview of available mathematical models of spatial virus spread

Model	Modelling formalism	Key assumptions	Predictions	Applications
HIV propagation in space and in time [1]	3D stochastic cellular automata	Diffusion-limited propagation of HIV by infected CD4+ cells	Excitable medium type of behavior. Stability- and geometry limited regimes of viral spread. Propagations regimes (1) travelling wave, (2) chaotic steady state, (3) extinguishing of infection	Relevant for in vitro system as the effect of CD8+ T T cell is not considered
VSV growth and spread [2]	Set of reaction-diffusion PDEs, ODEs, Integro-differential PDEs	2D radial symmetry, infection of kidney cells, IFN response	Estimated of the parameters of VSV-BHK infection growth and spread	In vitro studies of spatially spreading viruses over multiple generations characterized by immuno-histochemical labeling and digital imaging
IAV infection of humans [3, 4]	2D square lattice cellular automation [3]; two-dimensional sheet of hexagonally -tilled epithelial cells [4]	Infection grows locally around infected site by spreading from cell-to-cell	Distribution of initially infected cells has a great impact on the infection dynamics, the impact of the local versus global regeneration rules, the importance of occasional jumps in viral spread to previously uninfected areas	Target-cell limited resolution of infection, sustainable spread of infection as a propagating wave, low level (2% of infected cells) persistence of infection [3]

Model	Modelling formalism	Key assumptions	Predictions	Applications
Multi-compartment virus-target cell-immune cell dynamics with a local random spread of the virus [5]	System of ODEs for basic virus-immune dynamics at 21x21 site grid	Sessile target and infected cells. Spatial coupling by nearest neighborhood dispersal of the virus, predator-prey type of virus-immune dynamics	A robust persistence of infection for intermediate dispersal rate; rejected the “dynamic elimination” paradigm	Analysis of HIV and SIV dynamics around the viral set point
Dynamics of HIV-1 infected target cells [6]	System of distributed DDEs	Cell-to-cell spread of HIV-1, well-mixed system	Existence of sustained oscillations of infection for typical tissue culture parameters	Key role of latently infected cells in sustaining the infection, HIV persistence in the form of infective oscillations
Spatiotemporal dynamics of virus - target cell system in HIV infection [7]	System of PDEs describing infection, replication, diffusion and chemotaxis	2D surface domain, purely Brownian motion of cells, bounded rate chemotactic movement	The speed of the pattern propagation, the spatial frequency and amplitude of the density variation (hot spots)	Initial foci of HIV infection can be established as a result of the virus-target cell dynamics; immune system response may give rise to pattern formation

Model	Modelling formalism	Key assumptions	Predictions	Applications
Viral infection in spherical organs [8]	System of reaction-diffusion equations with influx boundary conditions and radial symmetry	3D view of infected organ with a spherical shape, viruses and immune cells penetrate organ at the surface and propagate to the inner domain	Analytical solutions showing non-uniform distribution of viruses and cells in the organ; the role of diffusivity parameters; temporal and spatial profiles of the infection evolution	Areas of high viral concentration in organs; lack of accessibility of some inner parts of the organs for the immune cells and antiviral drugs
Virus growth in vitro [9]	System of master equations for the evolution of the number of infected cell in foci for HCV	Cell-to-cell mode of viral spread in the in vitro culture; no cell death; empirical density function for the time to infection of the founding cell in a focus	Growth of larger foci is best explained by the birth model; growth of the smaller foci is better described by the boundary model	Quantitative analysis of the efficacy for blocking HCV cell-to-cell spread when targeting different host factors

References

1. Strain MC, Richman DD, Wong JK, Levine. Spatiotemporal dynamics of HIV propagation. J Theor Biol. 2002, 218(1):85-96.

2. Haseltine EL, Lam V, Yin J, Rawlings JB. Image-guided modeling of virus growth and spread. Bull Math Biol. 2008, 70(6):1730-48

3. Beauchemin C. Probing the effects of the well-mixed assumption on viral infection dynamics. J Theor Biol. 2006, 242(2):464-77.

4. Levin D, Forrest S, Banerjee S, Clay C, Cannon J, Moses M, Koster F. A spatial model of the efficiency of T cell search in the influenza-infected lung. J Theor Biol. 2016, 398:52-63

5. Funk GA, Jansen VA, Bonhoeffer S, Killingback T. Spatial models of virus-immune dynamics. J Theor Biol. 2005, 233(2):221-36.

6. Culshaw RV, Ruan S, Webb G. A mathematical model of cell-to-cell spread of HIV-1 that includes a time delay. J Math Biol. 2003, 46(5):425-44.

7. Stancevic O, Angstmann CN, Murray JM, Henry BI. Turing patterns from dynamics of early HIV infection. Bull Math Biol. 2013, 75(5):774-95.

8. Dunia R, Bonnecaze R. Mathematical modeling of viral infection dynamics in spherical organs. J Math Biol. 2013;67(6-7):1425-55.

9. Graw F, Martin DN, Perelson AS, Uprichard SL, Dahari H. Quantification of Hepatitis C Virus Cell-to-Cell Spread Using a Stochastic Modeling Approach. J Virol. 2015;89(13):6551-61.
